# The aftermath of terrorism: posttraumatic stress and functional impairment after the 2011 Oslo bombing

**DOI:** 10.3389/fpsyg.2015.01156

**Published:** 2015-08-07

**Authors:** Øivind Solberg, Ines Blix, Trond Heir

**Affiliations:** ^1^Norwegian Centre for Violence and Traumatic Stress StudiesOslo, Norway; ^2^Institute of Clinical Medicine, Faculty of Medicine, University of OsloNorway

**Keywords:** post-traumatic stress disorder, functional impairment, terrorism, exposure, emotional numbing

## Abstract

**Objective:** In the present study we wanted to investigate the link between exposure, posttraumatic stress symptomatology, and functional impairment in the aftermath of terrorism.

**Method:** Posttraumatic stress symptomatology and functional impairment related to the Oslo bombing 22nd of July, 2011, in directly and indirectly exposed individuals (*N* = 1927) were assessed together with demographics, exposure, peri-traumatic reactions, and event centrality approximately 1 year after the attack.

**Results:** Directly and indirectly exposed individuals qualifying for posttraumatic stress disorder (PTSD) reported similar peri-traumatic reactions, event centrality, and functional impairment. However, clusters within the PTSD symptomatology were differentially associated with impairment as a function of their exposure. In the directly exposed group, all clusters within the PTSD symptomatology were associated with impairment in function, while only emotional numbing was associated with impairment within the indirectly exposed group.

**Conclusion:** Considering that terror attacks frequently involve directly exposed individuals and a larger population of indirectly exposed individuals, this finding is of importance, especially in the design of intervention programs and the development of treatment policies.

## Introduction

The link between exposure to terrorism and posttraumatic stress is well-documented (North et al., 1999, 2011; Galea et al., 2002; Rubin et al., 2005; Gross, 2006; Laugharne, 2007; Brewin et al., 2008; Bowler et al., 2012; Hansen et al., 2013; Holman et al., 2014). Across studies, data on exposure and risk consistently show that 25–40% of people affected by terrorism develop posttraumatic stress disorder (PTSD), with close proximity and/or direct exposure as a significant predictor of mental health disruption (Galea et al., 2002; Schlenger et al., 2002; Jordan et al., 2004; Rubin et al., 2005; Gross, 2006; Gabriel et al., 2007; Laugharne, 2007; Park et al., 2008; DiGrande et al., 2011; Hansen et al., 2013). However, a number of studies have also shown that terrorist attacks can have widespread mental health effects, even in geographically distant areas. In fact, in their investigation of the World Trade Centre attack, Schuster et al. (2001) found that Americans across the country had substantial stress reactions, even in remote regions. In a similar vein, North et al. (2011) showed that PTSD symptom criteria were met by 35% of those exposed only through a close associate’s direct exposure, while Holman et al. (2014) found that significant negative mental health effects can spread beyond the directly affected population through media exposure (Holman et al., 2014).

In an effort to explain these findings, Rubin et al. (2008) posited a theoretical mnemonic model of PTSD. According to this theory an interaction between exposure and the processes of remembering determines whether posttraumatic stress symptoms develop. By replacing the central role played by the event per se with the event as reconstructed in memory, predisposing personality and demographic factors are ascribed an active role in the interaction. Predisposing factors are thought to influence both the initial perception of the event and the event as construed in memory (Rubin et al., 2008). It follows that both direct and indirect exposure to trauma can lead to PTSD symptom development since it is the individual’s constructed memory of the traumatic event, and not the event itself that determines trauma symptoms. Still, direct exposure involving, e.g., violence and a direct threat to oneself, will interact with the processes of remembering in a different manner than indirect exposure to the same event will do. This might in turn explain why direct exposure to a traumatic event and the following symptom sequela often has a more debilitating effect on every day function.

However, previous efforts to bridge the gap between differential exposures, PTSD symptomatology and impairment in function have so far been inconclusive (Maguen et al., 2007), and the differential impact of symptom clusters within the PTSD diagnosis on function is still debated (Maguen et al., 2009; Heir et al., 2010). Indeed, findings from the Oklahoma City bombing showed that only avoidance symptoms had significant associations with functional impairment, while the hyper-arousal symptom clusters did not (North et al., 1999). In contrast, in their investigation of the 2004 South–East Asian tsunami, Heir et al. (2010) found that hyper-arousal symptoms, compared to the other PTSD symptom clusters, were most closely linked to functional impairment, with the avoidance symptom cluster exhibiting the smallest association. Finally, several studies have singled out symptoms of emotional numbing as the key predictor of functional impairment (e.g., Breslau et al., 2005; Miller et al., 2008) and even when re-experiencing and hyperarousal symptoms are more prevalent, less frequent emotional numbing symptoms have been shown to exhibit stronger associations with functional impairment (North et al., 1999).

In light of the aforementioned, exposure, together with individual symptomatology profiles, deserves careful consideration when investigating the link between posttraumatic stress and functional impairment. In the present study, we therefore wanted to investigate the link between direct and indirect exposure, posttraumatic stress symptomatology and functional impairment in the aftermath of the 2011 Oslo bombing. In line with Rubin et al.’s (2008) mnemonic theory, we hypothesized that posttraumatic stress and levels of impairment would differ according to exposure, with directly exposed individuals reporting higher symptom levels and more severe impaired function than indirectly exposed individuals. We also hypothesized that heightened functional impairment would be most closely linked to posttraumatic stress symptoms found in the hyperarousal and emotional numbing clusters, regardless of exposure type.

## Materials and Methods

### Data Collection and Participants

This study is part of a lager project conducted by the Norwegian Centre for Violence and Traumatic Stress Studies which focuses on the terrorist attack that targeted Norwegian government employees on the 22nd of July, 2011 (see Hansen et al., 2013 for details). Data were collected through a secure web-based questionnaire 9–10 months after the terrorist attack.

The study population comprised all individuals who were employed in 14 of the 17 Norwegian ministries on the 22nd of July 2011(*n* = 3579). All participants were informed about the study by their respective ministries through internal meetings and emails. All invited employees received a project specific identification number based on their social security number and a personalized code to log on to the questionnaire. 59 employees could not be reached with information about the study and 1550 did not respond to the questionnaire (Response rate = 56%). Due to missing data on key variables (e.g., PCL), 43 additional respondents were lost, leaving a final sample of 1927 for the present study.

An administrative person, who was not a member of the research group, matched the project ID number with the corresponding social security number, thus keeping the identity of respondents anonymous to the researchers. The study was approved by the Regional Committee for Medical and Health Research Ethics, and all participants were informed about the purpose and content of the study and the opportunity to withdraw.

### Measures

#### Demographics

Demographic data, including gender, age, and educational level were collected. Educational level was divided into categories of low, mid, and higher education, corresponding to “< 13 years,” “13–16 years,” and “> 16 years”, respectively.

#### Direct and Indirect Exposure

Exposure to the actual site or epicenter of the explosion was assessed by asking employees where they were located when the bomb went off, using five exposure categories: (1) “in the government district downtown,” (2) “in downtown Oslo, but not in the government district,” (3) “in Oslo, but not downtown,” (4) “in Norway, but not in Oslo,” and (5) “abroad.” These categories were subsequently collapsed into two categories (1 and 2–5) reflecting direct and indirect exposure to the epicenter (DirEx and IndirEx).

#### Symptom Checklist (SCL-6D)

The Hopkins Symptom Checklist-10 (SCL-10), Norwegian version (Tambs and Moum, 1993; Strand et al., 2003), incorporates two subscales, four and six items, allowing for separate analyses of depression and anxiety scores (SCL-6D and SCL-4A). In the present study the SCL-6D was used to control for comorbid symptoms of depression. Symptoms include, e.g., self-blame, sleep disruption, and feelings of low self-worth. Short-form versions of SCL have previously been shown to correlate highly with the total score of the original scale and to have good psychometric properties (Tambs and Moum, 1993; Strand et al., 2003; Solberg et al., 2011), measuring symptoms of depression almost as well as the full scale. Items in the SCL are scored on a Likert scale ranging from 1 (not at all bothered) to 4 (very much bothered) and, in the present study, Cronbach’s alpha for SCL-10 were 0.92. A mean score above 1.85 is regarded as a valid predictor of depression (Tambs and Moum, 1993).

#### Posttraumatic check list (PCL)

PTSD symptoms were assessed using a Norwegian version (Hem et al., 2012) of the original PCL (Weathers et al., 1993). The PCL is a 17-item self-administered questionnaire that assesses the full PTSD domain described in the diagnostic and statistical manual fourth edition (APA, 1994). In the present study posttraumatic stress reactions were measured with PCL-S (Weathers et al., 1993; Blanchard et al., 1996; Forbes et al., 2001). In this version the symptoms endorsed are specifically linked to a traumatic event and instructions to consider the Oslo bombing, 22nd of July, 2011 as reference point when answering were given. In the PCL respondents are asked to what degree they have been bothered by each symptom (e.g., unpleasant memories, nightmares, sleep disturbances etc.) in the previous month on a scale ranging from 1, “not at all” to 5, “extremely,” with a total score of 85. In order to distinguish between a PTSD group and a non-PTSD group we applied the DSM-IV criteria on the PCL responses (Hem et al., 2012). Items with a score of three or higher were counted as symptoms, and a probable PTSD diagnosis was then determined by following the DSM-IV criteria, which requires at least one re-experiencing symptom (Criterion B), at least three avoidance/numbing symptoms (Criterion C), and at least two hyperarousal symptoms (Criterion D).

The Norwegian version of the scale has been shown to perform well as a diagnostic instrument for detecting PTSD in the Norwegian population (Hem et al., 2012). In the present sample the internal consistency of the PCL was high with a Cronbach’s alpha of 0.94 for the total scale and 0.86, 0.86, and 0.89 for the three subscales, respectively. Sub-scale symptom clusters within PCL were grouped according to DSM-IV criteria corresponding to criterion B/Intrusions (item 1–5), criterion C/Avoidance (item 6–12), and criterion D/Hyperarousal (item 13–17). Furthermore, criterion C was divided into avoidance (6–7) and numbing (8–12) clusters according to factor structure recommendations (King et al., 1998; Palmieri et al., 2007; Scher et al., 2008; Maguen et al., 2009).

#### Centrality of Event Scale (CES)

The CES measures the extent to which a memory for a traumatic event forms a reference point for personal identity and for the attribution of meaning to other experiences in a person’s life (Berntsen and Rubin, 2006). The short version is a 7-item questionnaire with items rated on a scale from 1 (strongly disagree) to 5 (strongly agree). The scores are calculated as the mean of all items with high scores indicating high centrality. The original version and the Norwegian short 7-item version has been reported to be reliable (α = 0.88–92; Berntsen and Rubin, 2006; Blix et al., 2014).

#### Peri-Traumatic Reactions

Immediate or peri-traumatic reactions were measured in relation to the DSM-IV A2 criterion; “The person’s response involved intense fear, helplessness, or horror,” using three questions in Norwegian asking whether employees experienced fear, helplessness, and horror on a scale from 1 (not at all) to 5 (to a high degree).

#### Work and Social Adjustment Scale (WSAS)

In an effort to assess the burden of PTSD symptomatology, a five-item scale of functional impairment attributable to an identified problem/disorder was utilized (Mundt et al., 2002). According to the authors the WSAS exhibit strong psychometric properties (Cronbach’s α = 0.79–94) across several studies. In the present sample the internal consistency of a Norwegian version of WSAS was high with a Cronbach’s alpha of 0.96. Items include statements such as, e.g., “Because of my disorder my ability to work is impaired” and/or “Because of my disorder my social leisure activities (with other people, such as parties, bars, clubs, visits, dates, and home entertainment) are impaired.” Scores range from (0) “not at all impaired” to (8) “very severely impaired,” with a total score of 40. According to Mundt et al. (2002) a WSAS score of 20 or above indicates moderately severe or worse psychopathology, whereas scores below 10 is to be associated with subclinical populations. Scores between 20 and 10 suggest significant impairment, but less severe symptomatology.

### Statistical Analyses

All analyses were conducted using data collected from web-based questionnaires completed by Government employees who were on the pay-roll at the time of the attack. In order to examine the impact of exposure and PTSD on functional impairment (WSAS scores), the sample was divided into four vulnerability groups reflecting proximity and diagnostic status: (1) “IndirectEX/No PTSD”, (2) “DirectEx/No PTSD”, (3) “IndirectEx/PTSD,” and (4) “DirectEx/PTSD.”

Means and frequencies were calculated for demographic information, predictors and dependent variables. All measures were checked for score distributions (skewness and kurtosis) and homogeneity of variance using Levenes test. We also tested for multicollinearity to make sure that the model estimates of the coefficients weren’t unstable and/or the standard errors for the coefficients inflated. As with most measures assessing health disruption, responses on posttraumatic stress (PCL), functional impairment (WSAS) and depression (SCL-D) were slightly skewed in all groups. Still, Levenes tests for the PTSD groups were not significant (PCL; *F* = 0.359, *p* = 0.550, WSAS; *F* = 0.674, *p* = 0.414, SCL-D; *F* = 0.003, *p* = 0.958) indicating equal variances and a collinearity diagnosis revealed acceptable values (tolerance >0.43 and VIF <2.31).

Differences on WSAS scores between the four vulnerability groups were then assessed using a one-way analysis of variance with Bonferroni adjusted confidence intervals and covariates. Multiple linear regression analyses were also conducted to identify symptom clusters within the PTSD symptomatology (PCL) associated with WSAS severity across groups. Finally, the PROCESS add-on for SPSS was used to test for possible mediation processes within our cross-sectional design using the “Indirect exposure/Direct exposure” measure as the independent variable (*X*), mean PCL score (posttraumatic stress) as the mediator (*M*) and mean WSAS scores (functional impairment) as the outcome variable (*Y*). The procedure uses an ordinary least squares or logistic regression-based path analytical framework for estimating direct and indirect effects in simple and multiple mediator models (Hayes, 2013; Hayes and Scharkow, 2013). See **Table 1** in the results section for coverage of the sample characteristics.

**Table 1:** Characteristics of the sample.

Characteristics	Direct exposure		Indirect exposure	
	PTSD (*n* ≈ 49)	No PTSD (*n* ≈ 155)	PTSD (*n* ≈ 64)	No PTSD (*n* ≈ 1659)
Age (years) *M* ± SD	43.7 ± 11.70	45.0 ± 11.93	47.1 ± 11.00	45.4 ± 10.80
Gender (female %)	81.6^†^	54.2	70.3^†^	56.8
Education (low/mid/high %)	10.2/36.7/53.1	11.6/28.4/60.0	20.3/34.4/45.3	11.1/26.3/62.6
SCL-6D *M* ± SD	2.52 ± 0.72^∗∗^	1.32 ± 0.39	2.58 ± 0.69^∗∗^	1.28 ± 0.44
CES-7 *M* ± SD	3.38 ± 0.87^∗∗^	2.02 ± 0.85^∗∗^	3.17 ± 1.04^∗∗^	1.67 ± 0.73
Peri-traumatic reactions *M* ± SD	11.35 ± 2.32^∗∗^	8.9 ± 2.91^∗∗^	12.05 ± 2.29^∗∗^	9.65 ± 2.71
Posttraumatic stress (PCL-17) *M* ± SD	55.80 ± 10.54^∗^	27.15 ± 7.82^∗^	51.58 ± 9.64^∗^	22.39 ± 6.56
*B/Re-experiencing/Intrusions M* ± SD	3.24 ± 0.76*^∗^*^∗^	1.63 ± 0.63*^∗^*^∗^	3.02 ± 0.79*^∗^*^∗^	1.37 ± 0.48
*C/Avoidance M* ± SD	3.12 ± 1.08*^∗^*^∗^	1.57 ± 0.67*^∗^*^∗^	3.01 ± 1.01*^∗^*^∗^	1.33 ± 0.61
*C/Numbing M* ± SD	2.89 ± 0.79*^∗^*^∗^	1.36 ± 0.44*^∗^*^∗^	2.77 ± 0.70*^∗^*^∗^	1.15 ± 0.32
*D/Hyperarousal M* ± SD	3.78 ± 0.74^∗∗^	1.80 ± 0.70^∗∗^	3.32 ± 0.72^∗∗^	1.42 ± 0.60


*^∗^P < 0.05; ^∗∗^P < 0.001, Compared to indirect/No posttraumatic stress disorder (PTSD) using analysis of variance (ANOVA) and Pairwise comparisons*.

^†^*Compared to indirect/No PTSD using Cross-tabulation with Chi-square tests*.

## Results

### Exposure, PTSD Diagnosis, Work and Social Adjustment (WSAS)

The univariate analysis of variance (ANOVA) revealed a main effect of group, [(1) IndirectEx/No PTSD, (2) DirectEx/No PTSD, (3) IndirectEx/PTSD, and (4) DirecrtEx/PTSD] on the dependent variable WSAS [*F*(3,1790) = 340.529, *p* < 0.000], which indicated that both exposure and the presence of PTSD were associated with impairment in WSAS. The DirectEx PTSD group reported a WSAS score of 21.91, SD = 8.94, while the IndirectEx PTSD group reported a score of 18.39, SD = 9.39, suggesting significant impairment. The non-PTSD groups (DirectEx/IndirectEx) on the other hand reported scores of 4.57, SD = 5.99, and 2.47, SD = 5.03, respectively, indicating subclinical impairment.

To test for a mediational path between exposure, PTSD symptoms and functional impairment, we used a bootstrap approach to mediation as suggested by Hayes (2013). The analysis, using 20,000 bootstrap samples, showed that there was an indirect effect of exposure on functional impairment (WSAS) through PCL levels (95% CI 0.855, 1.350, point estimate 1.082), while the direct effect was non-significant.

### Exposure, PTSD Symptom Clusters, WSAS

A multivariate analysis of variance (MANOVA) with between subjects effects (DirectEx/IndirectEx), including only employees with PTSD (*n* = 113), revealed no difference in mean scores for symptom cluster B/Intrusions, C/Avoidance, and Numbing between groups, but significantly higher scores within the D/hyperarousal cluster for the DirectEx group [*F*(1,104) = 10.432, *p* < 0.002]. See **Table 1** for group cluster means by group.

Next, a univariate ANOVA, again including only employees with PTSD, revealed no main effect of exposure, (DirectEx/IndirectEx) for the dependent variable WSAS [*F*(1,102) = 3.755, *p* < n.s.], indicating similar impairment in WSAS for both groups. Furthermore, a multiple linear regression analysis, again including only employees with PTSD, revealed that within the PTSD symptomatology, symptom clusters B/Intrusions, C/numbing, and D/Hyperarousal showed significant associations with WSAS scores, while the C/Avoidance cluster did not.

Interestingly, when applying a multiple linear regression analysis with the exposure split (DirectEx/IndirectEx), only the association between criterion C/numbing and WSAS scores reached significance in the IndirectEx group (Model 1), while all clusters in the DirectEx group reached significance. Furthermore, when controlling for symptoms of depression (SCL-6D), the significant association between C/numbing in the IndirectEx group disappeared, as shown in **Table 2**, Model 2 and 3.

**Table 2:** Multiple linear regression models.

	Indirect exposure PTSD			Direct exposure PTSD			
Model and Predictors	B (95%Cl)	β	*R*^2^	B (95%Cl)	β	*R*^2^
**Model 1**			0.42^∗∗^			0.80^∗∗^
Criterion B/Intrusions	0.434 (-0.215–1.084)	0.184		0.809 (0.348-1.271)	0.341^∗∗^	
Criterion C						
-Avoidance	-0.188 (-0.599-0.224)	-0.102		-0.389 (-0.663-0.116)	-0.237^∗^	
-Numbing	1.093 (0.453-1.733)	0.415^∗∗^		0.891 (0.496-1.287)	0.403^∗∗^	
Criterion D/Hyperarousal	0.541 (-0.163-1.246)	0.207		1.042 (0.552-1.532)	0.428^∗∗^	
**Model 2**			0.35^∗^			0.48^∗∗^
Depression (SCL-6D)	1.632 (1.037-2.227)	0.596^∗∗^		1.715 (1.156-2.274)	0.691^∗∗^	
**Model 3**			0.49^∗^			0.83^∗∗^
Depression (SCL-6D)	0.907 (0.218-1.596)	0.331^∗^		0.556 (0.046-1.065)	0.224^∗^	
Criterion B/Intrusions	0.373 (-0.245-0.990)	0.157		0.850 (0.408-1.292)	0.358^∗∗^	
Criterion C						
-Avoidance	-0.119 (-0.512-0.274)	-0.064		-0.451 (-0.718-0.184)	-0.274^∗^	
-Numbing	0.674 (-0.011–1.359)	0.256		0.686 (0.264–1.108)	0.310^∗^	
Criterion D/Hyperarousal	0.416 (-0.259-1.090)	0.159		0.830 (0.323–1.336)	0.341^∗^	

*Associations between PTSD symptom clusters and Work and Social Adjustment (WSAS) scores*.

*^∗^p ≤ 0.05, ^∗^p ≤ 0.001*.

*Model 1 Indirect exposure: F(4,52) = 9.56, p < 0.001, Model 1 Direct exposure: F(4,39) = 40.01, p <0 .001*.

*Model 2 Indirect exposure: F(1,55) = 30.25, p < 0.001, Model 2 Direct exposure: F(1,42) = 38.31, p < 0.001*.

*Model 3 Indirect exposure: F(5,51) = 9.93, p < 0.001, Model 3 Direct exposure: F(5,38) = 36.23, p < 0.001*.

## Discussion

In line with previous research and our hypotheses (Shea et al., 2010; Rodriguez et al., 2012), the present study showed that both exposure, diagnosis and higher symptoms scores of PTSD were associated with impairment approximately 10 months after the attack. Furthermore, in line with the findings of Miller et al. (2008), a bootstrap approach to mediation showed only an indirect effect of exposure through symptoms of PTSD, implying that the association between exposure and functional impairment might be fully mediated by PTSD severity.

However, when applying an exposure split (direct/indirect exposure), WSAS scores for the two PTSD groups were not statistically different, both indicating clinically significant impairment. Still, the directly exposed PTSD group reported significant associations between all symptom clusters and functional impairment, while only the C/numbing cluster reached significance for the indirectly exposed PTSD group. When adjusting for symptoms of depression, the C/numbing cluster failed to reach significance for the indirectly exposed PTSD group, whereas this adjustment did not affect the associations between PTSD clusters and function within the directly exposed group. These findings require further discussion.

### B-Criterion/Re-Experiencing/Intrusions

According to Rubin et al. (2008) it is the memory of the traumatic event, and not the event itself that determines trauma symptoms. In other words, it might be the content of intrusive trauma-memories that dominate the manifestation and maintenance of psychopathology in the aftermath of trauma. Our findings can be interpreted in line with this perspective. Although the level of intrusive symptoms reported by the directly exposed PTSD group was equal to the level reported by the indirectly exposed PSTD group, the phenomenology or content of the the most emotionally arousing moments themselves must contain very different images about the attack than those in the indirectly exposed group. This might in turn explain why these memories have a more profound impact on WSAS scores. Put in other words, re-experiencing a visually disturbing memory (e.g., scenes containing blood, smoke, and fire) from the actual explosion site might be more debilitating than re-experiencing a memory of, e.g., a disturbing phone call describing the same event.

### C-Criterion/Avoidance

In the present study, the C/Avoidance cluster was somewhat surprisingly inversely associated with functional impairment in the directly exposed PTSD group and, although not significant, the same pattern was observed in the indirectly exposed PTSD group. A similar inverse relationship between avoidance symptoms and functional impairment was also described in the study by Maguen et al. (2009) in post deployment Kosovo peacekeepers. Although this finding seems contra intuitive, avoidance in the sense of “not thinking or talking about the event” and “avoiding situations or activities that remind you of the event” may work in the short term, actually to some degree maintaining function at the workplace and at home, by “cloaking” intrusive memories and the anxiety that follows for the time being (Leaman et al., 2012).

Still, most cognitive models of posttraumatic stress propose that avoidant behavior is a primary reaction to intrusive thoughts/re-experiencing (Ehlers and Clark, 2000; Rubin et al., 2008), and only contributes to the persistence of intrusions (Wegner, 1994; Bryant and Harvey, 1995). The difference in the avoidance symptoms’ impact between the groups might therefore be explained as a function of the intrusive memories themselves. The contents of the intrusions might cause the directly exposed individuals to put more effort into their avoidance strategy compared to indirectly exposed individuals who has to cope with less disturbing intrusions. This, in turn, will affect function accordingly regardless of the frequency of the avoidant behavior. Considering the inverse relationship observed, one might speculate that employees who were present at the time of the attack, as a function of their intrusions, need to employ a more stringent avoidance strategy, for the time being reducing the events’ impact on their function.

### C-Criterion/Numbing

In our sample the C/numbing cluster evidenced the highest association with functional impairment in both PTSD groups. This is in accordance with the notion that emotional numbing appears to have especially deleterious effects on functional outcomes (Maguen et al., 2009). Moreover, research has shown that numbing symptoms can be used as a marker, distinguishing PTSD-diagnosed individuals from individuals only qualifying for partial/sub-threshold PTSD and/or no PTSD (Breslau et al., 2005). In fact, as mentioned in the introduction, North et al. (1999) found that although re-experiencing and hyperarousal symptoms were most prevalent, less frequent numbing, and avoidance symptoms exhibited the strongest associations with functional impairment. Furthermore, previous research (Litz, 1992; Tull and Roemer, 2003; Yoshihama and Horrocks, 2005; Palyo et al., 2008) have suggested that attempts to manage re-experiencing and hyperarousal symptoms deplete emotional resources, which in turn cause emotional numbing. Hence, in the vein of these thoughts and the mnemonic perspective, it is possible that both our PTSD groups expend cognitive, behavioral, and emotional efforts in an attempt to manage or avoid hyperarousal symptoms caused by traumatic memories of the event, exhausting or depleting their emotional resources. In turn this might lead to a lack of responsiveness and functional impairment in social and occupational domains.

But, why did the association between emotional numbing and function disappear in the indirectly exposed group and not in the directly exposed group when we controlled for depression? If we take into consideration that the hyperarousal scores reported by the directly exposed PTSD group were significantly higher than in the indirectly exposed PTSD group, it is fair to say that this higher level of hyperarousal should lead to higher demands on cognitive, behavioral, and emotional efforts in attempts to manage or avoid hyperarousal symptoms in this group. Since emotional numbing to some extent overlap with depression, we could speculate that the negative association between emotional numbing and function reported by both groups to some extent is made up by elements found in both emotional numbing and depression, but that more profound emotional numbing, as a result of heightened hyperarousal, also include elements not found in depression. Indeed, the notion of emotional numbing as a construct distinct from depression has received some support (Feeny et al., 2000) even though the interrelation between symptom criteria of emotional numbing (e.g., diminished interest in activities and restricted range of affect), and symptoms of depression is evident (Litz et al., 2002; Kashdan et al., 2006). Considering this, it is not surprising that a weak association between emotional numbing and functional impairment could be equally well explained using the concept of depression. On the other hand, it is also evident that a stronger association could consist of elements not found in depression, keeping an association significant although symptoms of depression explain some of the variance.

### D-Criterion/Hyperarousal

In our analyses the two PTSD groups only differed in their level of hyperarousal, with the directly exposed PTSD group reporting the highest scores. In line with the discussion of this paper, we propose that the observed difference in hyperarousal levels between the two groups is linked to their memories of the event. Put differently, the heightened arousal experienced by this group can be explained as a function of memory. The directly exposed group, who were present at the time of the explosion, experience memories containing more disturbing images than the indirectly exposed group. These disturbing memories, containing, e.g., recollections of the actual sound wave of the blast, fire, and smoke, produce pronounced hyperarousal, which in turn affect the development of symptomatology and impair function.

### Limitations

Main limitations include a moderate response rate of 56% and a cross-sectional design. Thus, we cannot exclude the possibility of sampling bias and only discuss possible causal/mediational directions. Further, the prevalence of PTSD was assessed by the PCL self-report inventory, which implies that our results must be interpreted with caution. However, the Norwegian version of PCL has been shown to perform well as a diagnostic instrument for detecting PTSD in epidemiological research. Moreover, a more comprehensive measure of impairment in function would have been desirable. Like so often, we assessed functioning in work and social domains rather than more comprehensively assessing functional outcomes (e.g., legal issues, financial functioning, problem drinking, violent behaviors, use of prescription drugs etc.) possibly leaving out important information that could have broadened the scope of our findings. Finally, our 10 months measurement point might seem arbitrary and it could be argued that we might have lost important information in regards to early symptom development. Still, symptom-level variability is known to be very high in the first months following a traumatic event, before stabilizing over time. Hence, if assessments are carried out too soon, we might only capture heterogeneous, common, and transient reactions that might naturally remit over the following months (Steenkamp et al., 2015). So, even though we might have lost important information in regards to early symptom development in the first few months after the terror attack, the present study might also have an advantage by placing the assessment 10 months after the attack, at the point where symptom fluctuations have stabilized.

## Conclusions and Implications

Previous research has shown that populations indirectly exposed to terrorism or disasters can report mental health consequences similar to those of directly exposed populations (Neria et al., 2008; North et al., 2011). Furthermore, it can be argued that it is the association between PTSD symptomatology and impairment in function that defines the true impact of the PTSD and the preceding trauma (Karsten et al., 2013). In the present study both directly and indirectly exposed employees, diagnosed with PTSD, reported equally significant functional impairment in the aftermath of the Oslo terrorist attack, July 22nd. In addition to this, a novel finding emerged. Clusters within the PTSD symptomatology were differentially associated with impairment as a function of exposure. Within the directly exposed PTSD group, all clusters were associated with impairment in function, while only emotional numbing was associated with impairment within the indirectly exposed PTSD group. Since terrorist attacks frequently involve both directly exposed individuals and vast populations that are indirectly exposed to the event, this finding is of importance.

The authors of the present study therefore want to emphasize the value of fully considering exposures when investigating impairment in the aftermath of a terrorist attack, especially when large, indirectly exposed populations are involved. Screening interventions should include a thorough evaluation of exposures and individual PTSD symptom profiles in relation to functional impairment in addition to criteria for a DSM-IV/5 diagnosis. Treatment should also be tailored accordingly. In accordance with our findings and discussion, we hypothesize that indirectly exposed individuals reporting symptomatology within the realm of a PTSD diagnosis could, to a lager extent, benefit from interventions/treatment targeting depression or depressive thoughts, while directly exposed individuals should benefit from a more memory focused therapy, targeting the most potent and frequently re-occurring memories of the event. Future research should explore this hypothesis further.

## Conflict of Interest Statement

The authors declare that the research was conducted in the absence of any commercial or financial relationships that could be construed as a potential conflict of interest.
